# P-1132. Antimicrobial Persistence for 10 Days After Antiseptic Skin Preparation in Healthy Volunteers

**DOI:** 10.1093/ofid/ofaf695.1326

**Published:** 2026-01-11

**Authors:** Tiffany Hanson, Tera Nordby, Dan Morse

**Affiliations:** Solventum, Maplewood, MN; Solventum, Maplewood, MN; Solventum, Maplewood, MN

## Abstract

**Background:**

Current guidelines recommend skin preparation with antiseptic agents to help reduce skin microbes and aid in the prevention of surgical site infections (SSIs) and catheter-related blood stream infections (CRBSIs). A recent study reported that skin preparation with a 2% chlorhexidine gluconate (CHG) + 70% isopropyl alcohol (IPA) agent led to reduced skin microbes in healthy volunteers that persisted for 7 days post-application.

**Methods:**

Two studies were performed where CHG/IPA sterile antiseptic solutions (SASs) were applied to the abdominal or inguinal region of healthy volunteers and allowed to dry before being covered with dressings. In the first study, the antimicrobial effects of two SASs, SAS1 and SAS2, were compared during a 4-day period post-application. In the second, the antimicrobial persistence of SAS1 was examined for 7 days or 10 days post-application. In both studies, the Log_10_ reduction in skin bacterial levels (CFU/cm^2^) from baseline was assessed.

**Results:**

The comparative study consisted of 21 males and 48 females with a mean age of 58.4 years old. The Log_10_ reduction in skin bacteria levels was similar over the 4-day period for SAS1 and SAS2, ranging from a mean Log_10_ reduction of 3.16 to 3.73 for the abdomen and 3.61 to 4.69 for the groin. The 10-day study with SAS1 consisted of 43 males and 22 females with a mean age of 41.7 years old. Application of SAS1 led to a mean Log_10_ reduction in bacteria on the abdomen of 2.75 at 10-minutes, 2.03 at 7-days, and 1.53 at 10-days post-application. The mean Log_10_ reduction in skin bacteria on the groin was 3.59 at 10-minutes, 2.80 at 7-days, and 2.42 at 10-days post-application. Overall, a single adverse event (AE; 1 of 135 Subjects) was observed; the AE was a mild rash, not serious, and resolved by the end of study after short-term corticosteroid treatment.

**Conclusion:**

Skin antiseptics comprised of 2% CHG and 70% IPA can be effective and safe antimicrobial skin antiseptics. In addition, the antimicrobial effects of SAS1 can be sustained for 10 days post-application when used on healthy volunteers. Further studies are needed to determine the antimicrobial persistence and effect on SSIs or CRBSIs when these antiseptic agents are used preoperatively or before catheterization procedures, respectively.

**Disclosures:**

Tiffany Hanson, Solventum: Employee Tera Nordby, n/a, Solventum: Employee|Solventum: Stocks/Bonds (Public Company) Dan Morse, MS, Solventum: Stocks/Bonds (Private Company)

